# SARS-CoV-2-Derived RNA Fragment Induces Myocardial Dysfunction via siRNA-like Suppression of Mitochondrial ATP Synthase

**DOI:** 10.3390/ijms26115392

**Published:** 2025-06-04

**Authors:** Shota Nukaga, Rina Fujiwara-Tani, Takuya Mori, Isao Kawahara, Ryoichi Nishida, Yoshihiro Miyagawa, Kei Goto, Hitoshi Ohmori, Kiyomu Fujii, Takamitsu Sasaki, Chie Nakashima, Yi Luo, Shiori Mori, Shingo Kishi, Ruiko Ogata, Hiroki Kuniyasu

**Affiliations:** 1 Department of Molecular Pathology, Nara Medical University School of Medicine, Kashihara 634-8521, Japan; 2 Division of Rehabilitation, Hanna Central Hospital, Ikoma 630-0243, Japan; 3 Department of Medical Ethics and Genetics, Kyoto University, Kyoto 606-8501, Japan; 4 Department of Cancer Biology, Institute of Biomedical Science, Kansai Medical University, Osaka 573-1010, Japan; 5 Department of Pathological Diagnosis, Nozaki Tokushukai Hospital, Daito 574-0074, Japan

**Keywords:** SARS-CoV-2, F1F0 ATP synthase, ATP5, siRNA, heart injury

## Abstract

Myocardial injury is a critical determinant of prognosis in severe acute respiratory syndrome coronavirus 2 (SARS-CoV-2) infection; however, its underlying mechanisms remain incompletely understood. In this study, we examined the effects of SARS-CoV-2-derived RNA fragments on human cardiomyocytes. We identified a 19-nucleotide sequence within the viral genome that shares complete sequence homology with the human F1F0 ATP synthase subunit alpha gene (ATP5A). This sequence was found to associate with Argonaute 2 (AGO2) and downregulate ATP5A expression via a mechanism analogous to RNA interference. Consequently, oxidative phosphorylation was suppressed in cardiomyocytes, leading to impaired myocardial maturation and the emergence of heart failure-like phenotypes. Notably, exosome-mimetic liposomal delivery of this RNA fragment to cardiomyocytes reproduced the ATP5A-suppressive effect. These findings suggest that SARS-CoV-2-derived RNA fragments may contribute to myocardial injury through the siRNA-like modulation of mitochondrial gene expression. Further validation in animal models and patient-derived materials is warranted.

## 1. Introduction

Severe acute respiratory syndrome coronavirus 2 (SARS-CoV-2), the causative agent of coronavirus disease 2019 (COVID-19), has resulted in a global pandemic with substantial morbidity and mortality [1,2]. The in-hospital mortality rate among SARS-CoV-2-positive patients has reached approximately 11%, increasing to 21% in individuals aged 75 years and older [3]. Among the complications associated with SARS-CoV-2 infection, “myocardial injury” has emerged as one of the most prevalent, affecting more than 7% of hospitalized patients and up to 22% of those in critical care settings [4,5,6].

Early clinical reports from China revealed heart failure in 24% of all infected individuals and in 49–52% of patients who succumbed to the disease [7,8]. Furthermore, 10.4% of infected patients experienced arrhythmias, and 2.8% developed congestive heart failure, a figure that increased to 24% among non-survivors [9]. Acute myocarditis has been documented in 0.2–0.4% of cases, even in the absence of pneumonia [10], while the psychosomatic burden of SARS-CoV-2 infection has been implicated in the onset of Takotsubo cardiomyopathy [11,12].

Despite these observations, the mechanisms underlying acute or decompensated heart failure in SARS-CoV-2 infection remain poorly defined [13]. A leading hypothesis suggests that myocardial injury is primarily caused by the direct infection of cardiomyocytes through the binding of the viral spike (S) protein to the angiotensin-converting enzyme 2 (ACE2) receptor [12,14,15,16]. ACE2 is abundantly expressed in type II alveolar epithelial cells, intestinal epithelial cells, vascular endothelial cells, and cardiomyocytes [15], rendering these cells vulnerable to viral entry and direct cytopathic effects that can result in viral myocarditis [17]. However, direct cardiac infection has been identified in only approximately 5% of cases and is not considered a major contributor to COVID-19–related mortality [10].

In addition to direct cytotoxicity, the infection of vascular endothelial cells via ACE2 can induce vasculitis and subsequent thromboembolic complications, such as myocardial infarction and stroke [12,18,19,20]. Progressive SARS-CoV-2 pneumonia often leads to a cytokine storm, which promotes systemic inflammation, hypoxemia, and thrombosis in the myocardium through excessive cytokine release [12,17,21,22]. These mechanisms are particularly exacerbated in patients with preexisting cardiovascular disease or diabetes, where chronic inflammation and endothelial dysfunction predispose to more severe cardiac injury and worse outcomes [12,23,24,25].

Despite the high incidence and prognostic significance of cardiac injury in SARS-CoV-2 infection, the direct effects of the virus on cardiomyocytes remain incompletely understood. This raises the possibility that indirect mechanisms, independent of direct viral invasion, may contribute to myocardial injury.

SARS-CoV-2 is a member of the coronavirus family and is classified as a positive-sense single-stranded RNA virus [26]. Its approximately 30-kilobase genome encodes four major structural proteins—spike (S), envelope (E), membrane (M), and nucleocapsid (N)—as well as a variety of accessory proteins [7,27]. During intracellular replication, negative-sense single-stranded RNA intermediates are synthesized as templates for genome replication [28,29,30]. Recent studies have suggested that these antisense viral RNAs can be incorporated into extracellular vesicles, such as exosomes, and subsequently released into the circulation, potentially mediating pathological effects in distant organs [31,32].

This concept has led to growing interest in the role of exosome-mediated transfer of viral components in the development of cardiovascular complications during SARS-CoV-2 infection [33,34]. However, the molecular constituents of these extracellular vesicles—and their biological relevance to myocardial injury—remain largely unexplored.

The myocardium is highly dependent on mitochondrial oxidative phosphorylation, which accounts for more than 90% of its ATP production [35]. Mitochondria occupy nearly one third of the cardiomyocyte volume and play a central role in maintaining myocardial bioenergetics. F1F0 ATP synthase, the terminal enzyme complex of the electron transport chain (ETC), is responsible for coupling the mitochondrial proton gradient to ATP synthesis. Dysfunction of ATP synthase has been implicated in the development of cardiomyopathy and heart failure [36,37]; however, the precise mechanisms linking mitochondrial injury to heart failure pathogenesis remain incompletely defined.

In this study, we investigated whether specific RNA fragments derived from the SARS-CoV-2 genome may act in a small interfering RNA (siRNA)-like manner to suppress ATP synthase expression in human cardiomyocytes. We hypothesized that this mechanism may contribute to mitochondrial dysfunction and myocardial injury in the context of SARS-CoV-2 infection.

## 2. Results

### 2.1. Identification of Homologous Sequences Between the SARS-CoV-2 and Human Genomes

The reference genome sequence of the SARS-CoV-2 Wuhan strain was retrieved from the NCBI Nucleotide database and subjected to homology analysis against the human genome using the NCBI BLAST algorithm. This analysis identified a 19-nucleotide sequence exhibiting two mismatches with the human ATP5A gene (Table 1). Comparable homologous sequences were also identified in the ATP5A orthologs of mouse and rat, containing two and three nucleotide mismatches, respectively, when aligned with the human sequence.

Further comparative analysis of additional SARS-CoV-2 strains revealed strain-dependent variability in this sequence. For example, the WA-PHL-001844 strain (USA) displayed three mismatches, whereas the Japan/sb_ncgms_cov_2_02761/2022 and LA-CDC-STM-94SR8FMQV/2022 strains showed complete identity with the human ATP5A sequence. Interestingly, an identical sequence was also detected in the genome of a non-SARS coronavirus strain, HKU1B.9 (SC2521).

### 2.2. Genomic Localization of the Homologous Sequence

**Figure 1:**
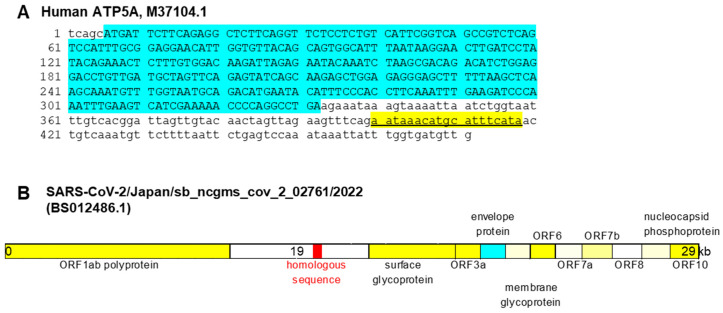
Location of ACHF. (**A**) ACHF in the ORF of ATF5A (yellow line) (Gene Bank accession number M37104.1). Blue line: ATP5A CDS. (**B**) ACHF in the Japanese SARS-CoV-2 (SARS-CoV-2/Japan/sb_ncgms_cov_2_02761/2022 (red mark) (Gene Bank accession number, BS012486.1). ACHF, ATF5A-CoV homologous fragment; ORF, open reading frame; ATF5A, ATP synthetase subunit 5A; CDS, coding sequence.

Next, we analyzed the genomic localization of the identified homologous sequence (Figure 1). In the human ATP5A gene, the sequence was located within an intronic region downstream of the coding sequence, yet still within the annotated open reading frame (ORF) (Figure 1A). In contrast, in the SARS-CoV-2 genome, the corresponding sequence was positioned between the ORF1ab polyprotein-encoding region and the spike (S) glycoprotein gene (Figure 1B).

Thus, in both the human and viral genomes, this sequence resides within non-coding regions. Based on this observation, we designated the sequence as the ATP5A–CoV Homologous Fragment (ACHF).

### 2.3. Danger-Associated Molecular Pattern (DAMP) Activity of ACHF

**Figure 2:**
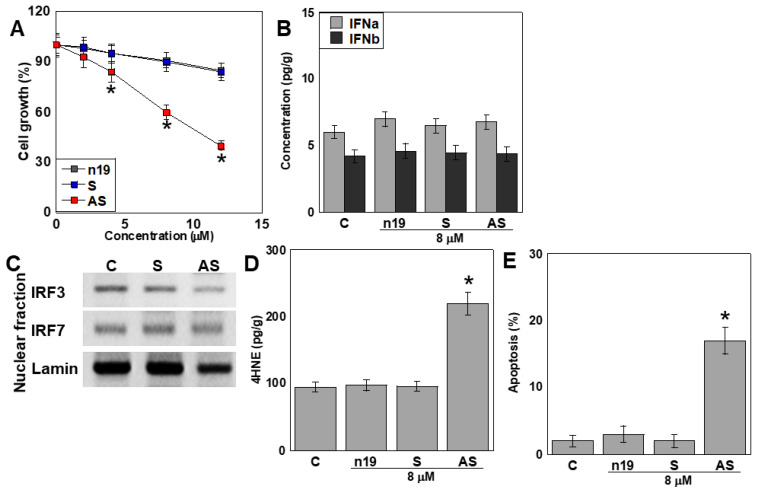
DAMP activity of ACHF in HCM. (**A**) Effect of the ACHF RNA fragment on HCM cell growth. (**B**–**E**) HCM were treated with 8 μM RNA fragments. (**B**) Intracellular type I IFN concentration. (**C**) Nuclear IRF3 and IRF7 in the RNA fragment-treated HCM. Lamin was used as the loading control. (**D**,**E**) 4HNE (**D**) and apoptosis (**E**) in the RNA fragment-treated HCM. Error bars represent the standard deviation from three independent trials. Statistical differences were calculated using standard ANOVA with Bonferroni correction. * *p* < 0.05 vs. n19 or C. DAMP, damage-associated molecular pattern; ACHF, ATF5A-CoV homologous fragment; HCM, human cardiac myocytes; S, sense sequence; AS, antisense sequence; n19, random 19 bases; C, untreated control; IFNa, interferon-α; IFNb, interferon-β; IRF, interferon regulatory factor; 4HNE, 4-hydrxynonenal; ANOVA, analysis of variance.

To investigate the potential DAMP-like activity of the ATP5A–CoV Homologous Fragment (ACHF), we synthesized single-stranded RNA (ssRNA) corresponding to the sense (S) and antisense (AS) strands of the ACHF sequence and applied them to human cardiomyocytes (HCMs) (Figure 2). Treatment with ACHF-AS resulted in a marked, dose-dependent inhibition of HCM proliferation (Figure 2A).

To assess the DAMP-associated immune response, we measured intracellular levels of interferon (IFN)-α and IFN-β following S or AS treatment. Neither treatment led to significant changes in IFN-α or IFN-β expression compared with untreated controls (Figure 2B).

We further examined the activation of pattern recognition receptor (PRR) pathways by evaluating the nuclear translocation of interferon regulatory factors IRF3 and IRF7, which are downstream of TLR3, TLR7, and TLR8—receptors known to detect ssRNA. However, no nuclear translocation of IRF3 or IRF7 was observed in response to either S or AS treatment (Figure 2C).

Interestingly, ACHF-AS treatment significantly increased intracellular oxidative stress (Figure 2D) and induced apoptotic cell death (Figure 2E), suggesting a non-canonical DAMP-like effect unrelated to classical TLR-IRF signaling.

### 2.4. Antisense Activity of ACHF

**Figure 3:**
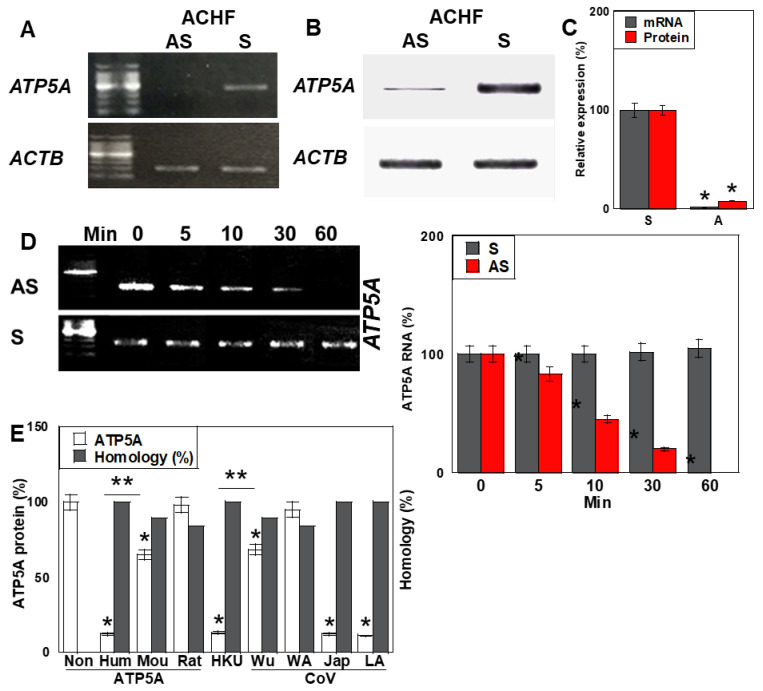
Suppression of *ATF5A* by ACHF in HCM. (**A**,**B**) Effects of ACHF on mRNA and protein levels of *ATP5A* in HCM. (**C**) Semi-quantification of panels (**A**,**B**). (**D**) Longevity of mRNA in ACHF-treated HCM. Right: semi-quantification of panel (**D**). (**E**) Differences in ATP5F protein repression among various ACHF sequences, and sequence homology between ACHFs and human *ATP5A*. Error bars represent the standard deviation from three independent trials. Statistical differences were calculated using standard ANOVA with Bonferroni correction. * *p* < 0.05 vs. S, ** *p* < 0.05. ACHF, ATF5A-CoV homologous fragment; ATF5A, ATP synthetase subunit 5A; HCM, human cardiac myocytes; S, sense sequence; AS, antisense sequence; CoV, SARS-CoV-2; ANOVA, analysis of variance.

To determine whether ACHF exerts antisense-mediated regulatory effects on its homologous gene, ATP5A, we evaluated ATP5A expression in human cardiomyocytes (HCMs) following treatment with either the sense (S) or antisense (AS) strands of ACHF ssRNA (Figure 3). ACHF-AS treatment resulted in a significant reduction in ATP5A mRNA levels compared to S treatment (Figure 3A,C). Correspondingly, ATP5A protein levels were markedly decreased in the AS-treated group (Figure 3B,C).

We next analyzed the temporal dynamics of ATP5A mRNA following AS and S treatment. In the AS-treated group, ATP5A mRNA levels gradually declined over time, while the S-treated group showed no significant change (Figure 3D).

To assess the sequence specificity of this suppression, we compared ATP5A protein levels in HCMs treated with ACHF sequences derived from different species and multiple SARS-CoV-2 strains (Figure 3E). Significant downregulation of the ATP5A protein was observed in cells treated with human-derived ACHF, as well as those from the HKU, Japan (Jap), and LA SARS-CoV-2 strains. Sequence alignment revealed that these ACHF variants shared 100% identity with the human ATP5A sequence. In contrast, ACHF variants from mouse and the Wuhan SARS-CoV-2 strain, each containing two nucleotide mismatches, reduced ATP5A expression by approximately 60% relative to the fully matched sequences. ACHF sequences from rat and the WA strain, which had three mismatches, induced only about 10% of the suppressive effect seen with the perfectly matched sequences.

These results indicate that the ACHF-AS RNA functions in a sequence-specific, siRNA-like manner, suppressing the expression of the homologous ATP5A gene in human cardiomyocytes.

### 2.5. Effects of the ACHF Sequence on Energy Metabolism

**Figure 4:**
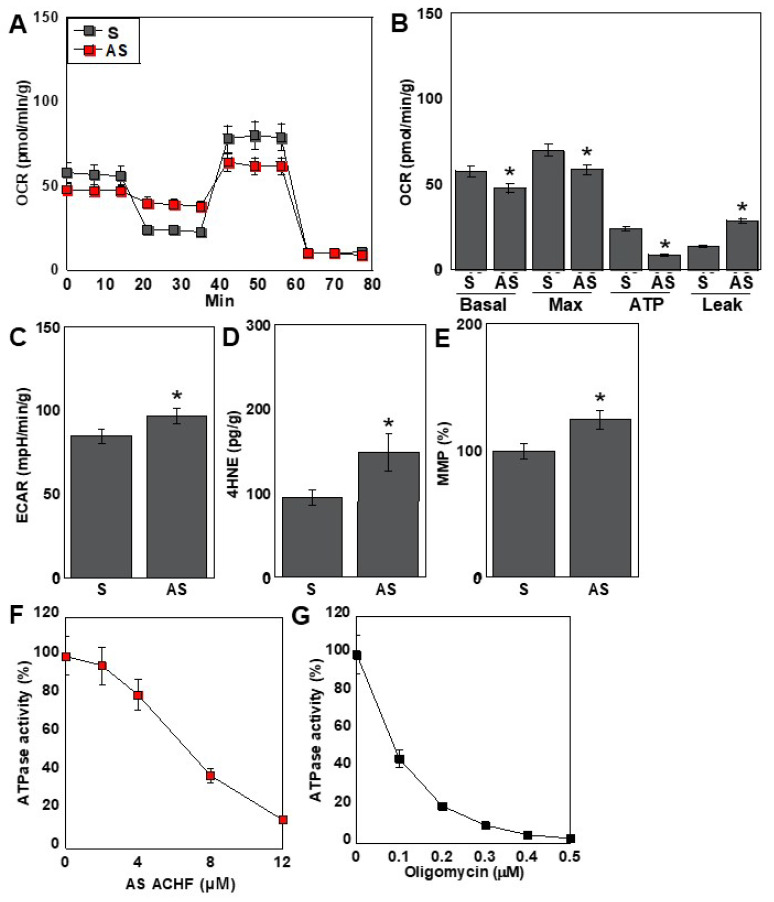
Effect of ACHF on energy metabolism in HCM. (**A**) Flux analysis in HCM treated with ACHF (4 μM). (**B**) Parameters in oxidative phosphorylation. (**C**) ECAR. (**D**) 4HNE. (**E**) MMP. (**F**) Effect of ACHF on F1F0 ATP synthetase activity. (**G**) Effect of oligomysine on F1F0 ATP synthetase activity. Error bars represent the standard deviation from three independent trials. Statistical differences were calculated using standard ANOVA with Bonferroni correction. * *p* < 0.05 vs. S. ACHF, ATF5A-CoV homologous fragment; ATF5A, ATP synthetase subunit 5A; HCM, human cardiac myocytes; S, sense sequence; AS, antisense sequence; OCR, oxygen consumption rate; ECAR, extracellular acidification rate; 4HNE, 4-hydroxynonenal; MMP, mitochondrial membrane potential; ANOVA, analysis of variance.

Given that the antisense (AS) strand of ACHF RNA suppressed ATP5A expression, we further investigated its impact on cellular energy metabolism in human cardiomyocytes (HCMs) (Figure 4). Metabolic flux analysis revealed a significant reduction in overall respiratory activity in the AS-treated group compared to the S-treated group (Figure 4A). Mitochondrial respiration parameters, including both basal and maximal respiration, were markedly decreased following AS treatment. Notably, intracellular ATP levels were significantly reduced, primarily due to an increase in proton leakage across the mitochondrial membrane (Figure 4B).

In contrast, glycolytic activity—as indicated by extracellular acidification rate (ECAR)—showed a modest increase in the AS-treated group (Figure 4C), suggesting a partial compensatory shift toward glycolysis. Oxidative stress levels, measured by intracellular 4-hydroxynonenal (4-HNE), were significantly elevated, while mitochondrial membrane potential (MMP) exhibited a slight increase (Figure 4D,E).

To further evaluate the mechanism underlying ATP depletion, we assessed the activity of the F1F0 ATP synthase complex. ACHF-AS RNA inhibited F1F0 ATPase activity in a dose-dependent manner (Figure 4F). To compare this inhibition with that of oligomycin, a well-established F1F0 ATPase inhibitor, we evaluated enzyme activity under matched conditions. The ACHF-AS RNA concentration used in the flux assay (0.4 µM) produced a comparable level of ATPase inhibition to that observed with 0.05 µM oligomycin (Figure 4G).

Collectively, these findings indicate that ACHF-AS RNA impairs mitochondrial oxidative phosphorylation by targeting ATP5A expression and F1F0 ATPase activity, leading to energetic stress, increased oxidative damage, and altered mitochondrial function in cardiomyocytes.

### 2.6. Effects of the ACHF Sequence on Cardiomyocyte Maturation

**Figure 5:**
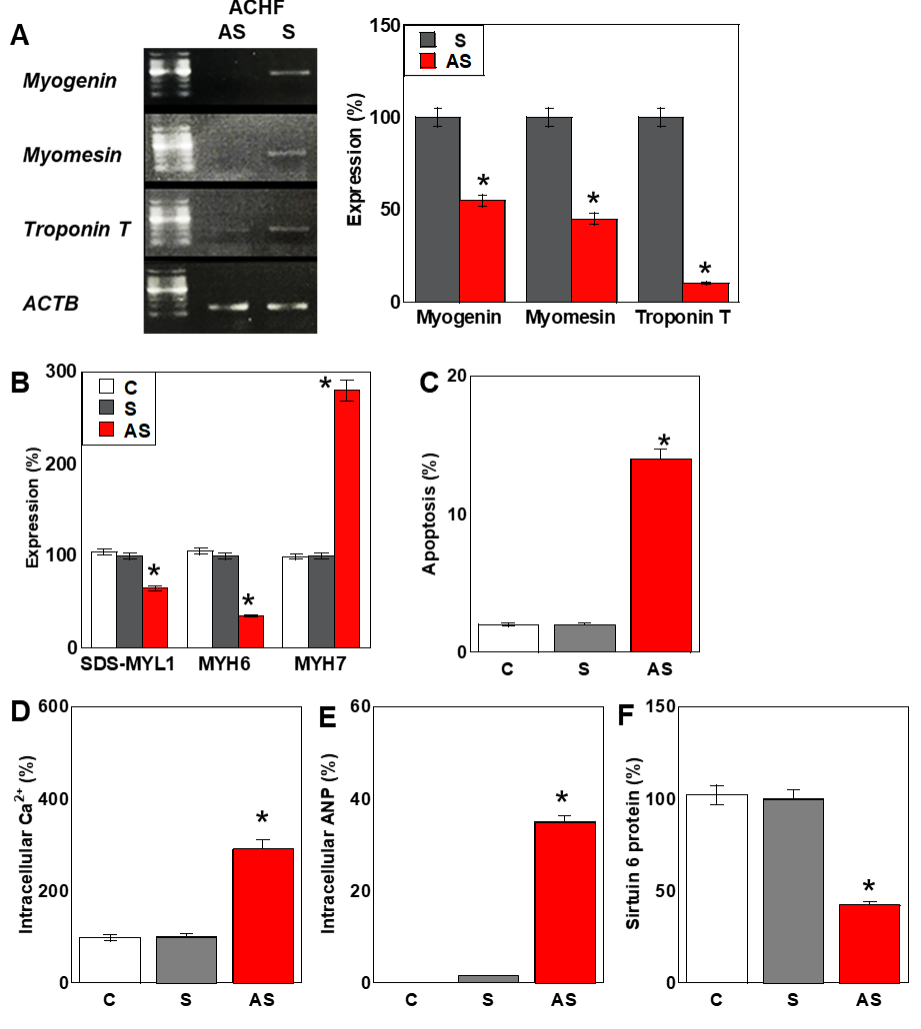
Effect of ACHT on maturation of HCM. (**A**) Expression of myocardial differentiation-associated genes. Right: semi-quantification of panel (**A**). (**B**) Levels of myocardial maturation-associated proteins. (**C**) Apoptosis. (**D**–**F**) Effect of ACHF on myocardial function: intracellular Ca^2+^ (**D**), intracellular ANP (**E**), and sirtuin 6 (**F**). Error bars represent the standard deviation from three independent trials. Statistical differences were calculated using standard ANOVA with Bonferroni correction. * *p* < 0.05 vs. S. ACHF, ATF5A-CoV homologous fragment; ATF5A, ATP synthetase subunit 5A; HCM, human cardiac myocytes; S, sense sequence; AS, antisense sequence; SDS-MYL1, sodium dodecyl sulfate-soluble myosin light chain-1; MYH, myosin heavy chain; ANP, atrial natriuretic peptide.

We next investigated the impact of ACHF antisense (AS) RNA on the maturation of human cardiomyocytes (HCMs) (Figure 5). In cells treated with ACHF-AS RNA, the expression of the early cardiomyocyte maturation markers myogenin and myomesin was reduced to less than 50% of that observed in the ACHF-S-treated group (Figure 5A). Moreover, troponin T, a marker of late-stage cardiomyocyte maturation, was decreased to approximately 10% of control levels.

We also analyzed the expression of myosin-related proteins associated with cardiomyocyte maturation. Protein levels of SDS-soluble myosin light chain 1 (SDS-MYL1) and myosin heavy chain 6 (MYH6) were decreased by 58% and 42%, respectively. In contrast, MYH7, a marker of immature myosin isoforms, was increased by approximately 280%, indicating a phenotypic shift toward an immature contractile profile (Figure 5B).

Consistent with these findings, the percentage of apoptotic cells increased from 1.6% in control cells to 14.7% in ACHF-AS-treated cells (Figure 5C). Intracellular calcium ion concentration, typically elevated in immature cardiomyocytes [38], was significantly increased in the AS-treated group (Figure 5D). Additionally, the level of atrial natriuretic peptide (ANP), a well-established marker of cardiac stress and heart failure, was elevated by approximately 50-fold (Figure 5E). The protein expression of sirtuin 6 (SIRT6)—which is downregulated in cardiac fibrosis [39]—was reduced by approximately 40% following AS treatment (Figure 5F).

These findings suggest that ACHF-AS RNA not only impairs energy metabolism but also disrupts cardiomyocyte maturation and promotes pathological remodeling, contributing to a phenotype resembling heart failure.

### 2.7. Mechanism of the Antisense Effect of ACHF-AS RNA

**Figure 6:**
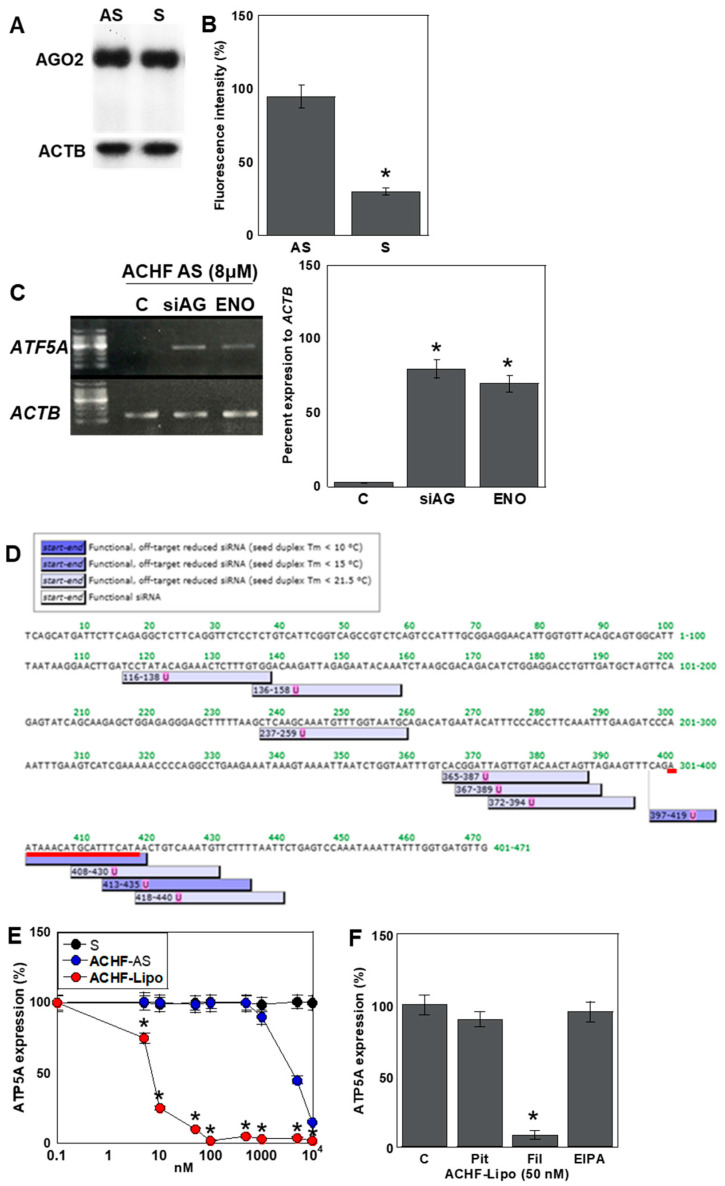
Mechanism of repression of ATP5A by ACHF in HCM. (**A**) Effect of ACHF on AGO2 protein. (**B**) Co-immunoprecipitation of biotin labeled-ACHF with AGO2 by evaluation of fluorescence. (**C**) Effect of AGO2 knockdown and enoxacin (20 μM) on ACHF-induced *ATP5A* repression. Right: semi-quantification of panel (**C**). (**D**) siRNA sequence designed for human *ATP5A* by siDirect software version 2.1. Red line, ACHF. (**E**) Effect of exosome-simulating liposome (Mikosome^®^) containing ACHF on ATP5A protein suppression. (**F**) Effect of inhibition of intracellular translocation of ACHF on ATP5A protein decrease using a clathrin-mediated endocytosis inhibitor, pitstop-2 (Pit, 20 μM); a caveolae-mediated endocytosis inhibitor, filipin (Fil, 3 μg/mL); and a macropinocytosis inhibitor, 5-(N-ethyl-N-isopropyl) amiloride (EIPA, 20 μM). Error bars represent the standard deviation from three independent trials. Statistical differences were calculated using standard ANOVA with Bonferroni correction. * *p* < 0.05 vs. S or C. ACHF, ATF5A-CoV homologous fragment; ATF5A, ATP synthetase subunit 5A; HCM, human cardiac myocytes; S, sense sequence; AS, antisense sequence; AGO2, argonaut 2; siAG, siRNA for AGO2; ENO, enoxacin; Lipo, liposome (Mikosome^®^); C, untreated control.

To elucidate the mechanism by which ACHF antisense (AS) RNA exerts its knockdown effect on ATP5A expression, we first examined the role of the RNA interference (RNAi) machinery (Figure 6). The expression level of Argonaute 2 (AGO2), a central component of the RNA-induced silencing complex (RISC), remained unchanged in both S- and AS-treated human cardiomyocytes (HCMs) (Figure 6A).

To assess AGO2 binding, we transfected HCMs with TAMRA-labeled S or AS ACHF RNA and quantified the fluorescence intensity of AGO2 immunoprecipitates (Figure 6B). ACHF-AS RNA showed approximately three-fold higher binding affinity to AGO2 compared to its sense counterpart, indicating preferential loading into the RISC.

Next, we tested the functional involvement of the RNAi pathway in ATP5A suppression (Figure 6C). The silencing of AGO2 or pharmacological inhibition of DICER using enoxacin abolished the knockdown effect of ACHF-AS RNA, demonstrating that the suppression of ATP5A is RISC-dependent.

To determine whether the ACHF-AS sequence functionally mimics a short interfering RNA (siRNA), we performed siRNA sequence prediction using siDIRECT. The results revealed that the ACHF sequence matches one of the predicted siRNA target sites for ATP5A, supporting its siRNA-like function (Figure 6D).

To simulate physiological conditions under which viral RNA fragments may be released from infected cells, we encapsulated ACHF-AS RNA in exosome-mimetic liposomes (Mikosome^®^) and delivered it to HCMs (Figure 6E). Notably, this method enhanced the suppressive effect on ATP5A expression by approximately 2000-fold compared to naked RNA transfection.

To identify the primary uptake mechanism of these liposome-encapsulated RNAs, we utilized pharmacological inhibitors of different endocytic pathways: pitstop-2 (clathrin-mediated endocytosis), filipin (caveolin-mediated endocytosis), and EIPA (macropinocytosis). Among these, filipin exhibited the most pronounced inhibitory effect, suggesting that caveolin-mediated endocytosis is the predominant route of cellular uptake for exosome-like vesicles (Figure 6F).

These findings suggest that ACHF may be released from SARS-CoV-2-infected cells encapsulated within exosomes and subsequently internalized by cardiomyocytes predominantly through caveolin-mediated endocytosis, where it exerts siRNA-like gene-silencing effects.

## 3. Discussion

In this study, we identified a 19-nucleotide sequence that is shared between the SARS-CoV-2 genome and an intronic region downstream of the coding sequence within the open reading frame (ORF) of the human ATP5A gene. We designated this sequence as the ATP5A–CoV Homologous Fragment (ACHF). Notably, antisense (AS) RNA corresponding to this sequence suppressed ATP5A expression, leading to mitochondrial respiratory dysfunction, impaired cardiomyocyte maturation, and increased apoptosis.

Mechanistically, ACHF-AS RNA exerts its gene-silencing effects through a small interfering RNA (siRNA)-like pathway mediated by Argonaute 2 (AGO2). Remarkably, this intronic sequence demonstrated siRNA-like activity targeting its own host gene. ACHF thus represents a rare example of a viral RNA fragment that shares homology with a non-coding region of the human genome and retains the potential to functionally regulate host gene expression.

Previous reports have shown that a substantial proportion of spliced introns escape complete degradation and are processed into various classes of non-coding RNAs (ncRNAs), including small RNAs, long non-coding RNAs (lncRNAs), and circular RNAs [40,41]. This observation raises the possibility that ACHF may function analogously to such host-derived ncRNAs.

The ability of ACHF to silence ATP5A expression is dependent on its binding to AGO2, the primary member of the human AGO protein family responsible for mediating RNA-induced gene silencing via both microRNAs (miRNAs) and siRNAs [42]. When an RNA sequence is loaded onto AGO2 within the RNA-induced silencing complex (RISC), the complex cleaves complementary target RNAs, resulting in a downregulation of protein expression [43].

Interestingly, ACHF shares full complementarity with the intronic region of ATP5A, and approximately 15% of known AGO2-binding sites are derived from intronic sequences [44]. Moreover, ACHF conforms to previously reported sequence characteristics that facilitate AGO2 binding, including optimal length, GC content, and the presence of a seed sequence [45]. These features likely enhance its ability to form a stable RNA–AGO2 complex and promote target RNA degradation [46,47]. Indeed, bioinformatic analysis using the siRNA design tool siDirect (http://sidirect2.rnai.jp/, accessed on 25 February 2025) confirmed that the ACHF sequence matches known siRNA sequences targeting ATP5A.

Taken together, these findings suggest that ACHF-AS RNA may bind to precursor or processed transcripts of ATP5A, thereby facilitating the targeted degradation and downregulation of gene expression. Although previous studies have shown that certain coronavirus-derived RNA fragments can influence host gene expression [48], the extent to which ACHF-AS RNA contributes to ATP5A suppression and cardiomyocyte injury in vivo remains to be determined. While extensive research has elucidated protein–protein interactions between host and viral factors, RNA–RNA interactions between viral RNAs and host transcripts remain largely unexplored, representing a novel and potentially impactful regulatory mechanism [48].

There are two possible scenarios by which ACHF may gain access to its target cells. In the first, ACHF is generated within SARS-CoV-2-infected cells and acts directly on the preprocessed ATP5A transcript before it undergoes splicing or further maturation. In the second, ACHF is produced in infected cells and subsequently released into the extracellular environment via exosomes or other extracellular vesicles, where it is taken up by uninfected bystander cells. Given that direct SARS-CoV-2 infection of cardiomyocytes is relatively infrequent [49], the latter scenario has attracted particular attention as a potential mechanism for indirect myocardial injury.

Antisense single-stranded RNA (ssRNA) is produced as an essential intermediate during the replication and transcription of the SARS-CoV-2 genome, which is a positive-sense ssRNA virus. This process is mediated by a virally encoded RNA-dependent RNA polymerase (RdRp) [28,29,30]. Emerging evidence suggests that viral antisense RNA intermediates are not merely passive templates but may possess intrinsic biological activity [50]. Notably, the SARS-CoV-2 replication cycle occurs entirely within the host cell cytoplasm, where viral RNAs coexist with a vast array of host RNAs [28]. This environment facilitates RNA–RNA interactions between viral and host transcripts, as seen in other viral infections: hepatitis C virus utilizes host miR-122 to enhance its replication [51]; Zika virus interacts with miR-21 [52]; and HIV-1 replication is primed by host-derived tRNAs [53]. These examples highlight the functional relevance of host–viral RNA interactions in modulating viral replication and host cellular responses.

The mechanisms underlying the release of SARS-CoV-2 RNA fragments are distinct from those of intact virions. While mature viral particles are typically exported through vesicle-mediated exocytosis, enabling direct infection of new target cells including cardiomyocytes [54,55], fragmented viral RNAs can be packaged into extracellular vesicles (EVs)—such as exosomes and microvesicles—and released into circulation [56]. These circulating RNA fragments have been increasingly implicated in multi-organ tissue damage and the pathogenesis of long COVID syndrome [57]. In our study, we demonstrated that exosome-mimicking liposomes encapsulating ACHF-AS RNA were efficiently internalized by cardiomyocytes and elicited potent siRNA-like effects. This finding supports a model in which ACHF-AS RNA, released from infected epithelial or immune cells in peripheral tissues, may travel systemically and modulate mitochondrial function in distant organs such as the heart.

Mechanistically, ACHF-AS RNA epigenetically suppresses ATP5A expression, leading to the inhibition of mitochondrial oxidative phosphorylation (OXPHOS) in human cardiomyocytes. OXPHOS is known to be regulated by a variety of miRNAs, several of which have been associated with the development of heart failure. For instance, miR-195 and miR-497 suppress NADH dehydrogenase 1 (Complex I) expression [58,59], while miR-210 targets cytochrome c oxidase subunits and iron–sulfur cluster assembly proteins [60]. Additionally, mitochondria harbor their own population of miRNAs, as well as AGO2 (encoded by the nuclear genome), which together regulate mitochondrial gene expression at the post-transcriptional level [61].

The suppression of ATP5A by ACHF-AS RNA thus represents a unique and previously unrecognized mechanism by which a viral RNA fragment, acting in an siRNA-like fashion, may impair mitochondrial electron transport chain (ETC) function and contribute to myocardial injury. This finding broadens the scope of known viral–host interactions by highlighting non-protein-coding, RNA-level crosstalk as a potential contributor to SARS-CoV-2 pathogenesis.

The human genome contains sequences derived from both DNA and RNA viruses [62]. Similarly, chimeric RNA species combining SARS-CoV-2 RNA and human genomic sequences have been reported [63,64]. RNA viruses can produce complementary DNA through reverse transcription, increasing the potential for integration into the host genome and the formation of endogenous retroelements [65,66,67]. Several regions of the SARS-CoV-2 genome are identical to human retroelement-derived sequences, including those corresponding to viral epitopes implicated in severe disease progression [68]. These viral epitopes share peptide sequence homology with human self-antigens, raising the possibility that molecular mimicry may trigger autoimmune responses contributing to the pathogenesis of severe COVID-19 [69,70,71,72]. Homology has been reported between SARS-CoV-2 proteins (e.g., spike, nucleocapsid) and host tissue antigens such as mitochondrial M2, F-actin, and thyroid peroxidase [73]. However, no significant sequence similarity has been identified between SARS-CoV-2 epitopes and mitochondrial electron transport chain (ETC) components such as ACHF. Moreover, ATP5A is a highly conserved, nuclear-encoded mitochondrial gene, making it unlikely to be retroelement-derived. These findings support the interpretation that the homology between ACHF and ATP5A is likely the result of random short-sequence similarity rather than evolutionary integration.

ACHF-AS RNA was shown to exert siRNA-like gene silencing activity, suppressing the expression of ATP5A, a core subunit of the F1F0 ATP synthase complex. This suppression inhibits OXPHOS and compromises cardiomyocyte function. The inhibition of mitochondrial OXPHOS impairs energy metabolism and reduces cardiomyocyte maturation. A decline in OXPHOS activity is a well-established contributor to heart failure through mechanisms including reduced myocardial contractility, elevated production of reactive oxygen species (ROS), and disruption of intracellular calcium homeostasis [74]. Importantly, myocardial injury in patients with SARS-CoV-2 infection—even in the absence of pre-existing cardiovascular disease—is associated with poor clinical outcomes [75]. While the direct viral invasion of cardiomyocytes has been observed in a limited number of cases, most histopathological studies reveal viral presence in interstitial tissues without signs of inflammation or myocyte infiltration [49]. These observations support the hypothesis that viral RNA fragments, rather than direct infection, may contribute to cardiac dysfunction in SARS-CoV-2 infection.

Although the circulating concentration of ACHF remains unknown, SARS-CoV-2 RNA has been detected in approximately 12.7% of early-phase serum samples from infected individuals, with RNAemia correlating with disease severity [76]. Furthermore, serum levels of troponin T, a biomarker of myocardial injury, increase in proportion to disease severity in COVID-19 patients [77]. These findings suggest that viral RNA fragments, including ACHF, may enter systemic circulation and exert biological effects on cardiomyocytes in severe cases.

To determine whether ACHF exhibits nucleic acid DAMP activity, we examined its ability to induce type I interferon (IFN-I) expression. Neither the sense (S) nor antisense (AS) strand of ACHF ssRNA activated IFN-α or IFN-β production, suggesting a lack of classical DAMP activity. Single-stranded RNAs can bind to endosomal Toll-like receptors (TLRs)—TLR7 and TLR8—initiating inflammatory responses via IFN production [78]. TLR7 preferentially recognizes uridine- or GU-rich sequences [79]. Although the uridine content of ACHF is 32% overall and 44% in the 3′ terminal nine nucleotides, ACHF lacks the UUGU motif, which is known to activate TLR7 and TLR8 [80]. Moreover, the activation of TLR3 typically requires double-stranded RNA structures of at least 40 base pairs in length [81], and secondary structure predictions showed that ACHF lacks such features. Thus, the observed effects of ACHF-AS RNA are likely attributable to antisense-mediated gene silencing, rather than immunostimulatory activity.

This study demonstrates that short homologous sequences shared between the viral and human genomes can function as antisense RNAs capable of modulating host gene expression. However, our findings are based on in vitro experiments, and several limitations must be acknowledged regarding their in vivo applicability. It remains unclear whether ACHF-AS RNA is generated in sufficient quantities from antisense replication intermediates during SARS-CoV-2 infection, and whether it is packaged into extracellular vesicles that can evade degradation and reach distant target tissues such as the heart. Further studies using animal models and patient-derived samples are essential to determine the biological relevance of ACHF in vivo and to validate its proposed role in SARS-CoV-2-associated myocardial injury.

## 4. Materials and Methods

### 4.1. Cell Line

Human cardiomyocytes (HCMs) derived from adult ventricular tissue were purchased from PromoCell (C-12810; Heidelberg, Germany). These cells display characteristics of cardiac progenitor cells and are not terminally differentiated. Cultures were maintained at 37 °C in a humidified incubator with 5% CO_2_, using Myocyte Growth Medium^®^ (D12028; TAKARA, Kyoto, Japan). The culture medium was refreshed every two to three days. When cultures reached 70–90% confluence, cells were detached using the Detach Kit (C-41200; TAKARA) and reseeded for further experiments.

### 4.2. Assessment of Cell Proliferation and Apoptosis

Cell viability was quantified using a tetrazolium-based colorimetric assay (MTS), employing the CellTiter 96 AQueous One Solution kit (Promega, San Luis Obispo, CA, USA). Absorbance at 490 nm was measured using a Multiskan FC microplate reader (Thermo Fisher Scientific, Waltham, MA, USA).

Apoptotic cells were identified by morphological criteria following Giemsa staining (Sigma-Aldrich, St. Louis, MO, USA), with a minimum of 1000 cells evaluated per sample to determine the percentage of apoptotic bodies.

### 4.3. Protein Isolation

Total protein was extracted as previously described [14], with minor modifications. Briefly, whole-cell lysates were obtained using radioimmunoprecipitation assay (RIPA) buffer supplemented with 0.1% SDS (Thermo Fisher Scientific, Tokyo, Japan). Subcellular fractionation was carried out using a commercial fractionation kit (Abcam, Cambridge, UK) following the manufacturer’s protocol. Protein concentration was determined using a colorimetric protein assay kit (Wako Pure Chemical Industries, Osaka, Japan).

### 4.4. Immunoblotting

Protein samples were separated by electrophoresis on 10% SDS–polyacrylamide gels and transferred to nitrocellulose membranes [82]. The membranes were probed with specific primary antibodies (listed in Table 2), followed by incubation with horseradish peroxidase-conjugated secondary antibodies (P0217; Dako, Glostrup, Denmark). Protein bands were detected using the Fusion Solo Imaging System (M&S Instruments Inc., Osaka, Japan).

### 4.5. Enzyme-Linked Immunosorbent and Fluorometric Assays

Quantitative protein analyses were conducted using ELISA kits to determine intracellular concentrations of 4HNE (ab287803, Abcam), IFN-α (ab213479, Abcam), IFN-β (DIFNB0, R&D Systems, Minneapolis, MN, USA), MYL1 (RK07660, Abclonal, Woburn, MA, USA), MYH6 (STJE0010826, St John’s Laboratory, London, UK), MYH7 (STJE0010828, St John’s Laboratory), and ATP5A (A310691, Antibodies.com, Cambridge, UK). Whole-cell lysates were prepared as described, and assays were performed according to each manufacturer’s protocol.

### 4.6. Reverse Transcription Polymerase Chain Reaction (RT-PCR)

Total RNA (0.5 μg) was isolated from cultured cells using the RNeasy Mini Kit (Qiagen, Germantown, MD, USA), and cDNA was synthesized for RT-PCR analysis. Primer sets, listed in Table 2, were designed and synthesized by Sigma Genosys (St. Louis, MO, USA). PCR products were resolved on 2% agarose gels and visualized with ethidium bromide under UV illumination. The housekeeping gene ACTB was amplified as a normalization control.

### 4.7. ATP Synthase Activity Assay

The enzymatic activity of the mitochondrial F1F0 ATP synthase (Complex V) was measured using a commercially available assay kit (Complex V Activity Assay Kit, #701000, Cayman Chemical Co., Ann Arbor, MI, USA) according to the supplier’s guidelines.

### 4.8. Assessment of Mitochondrial Membrane Potential (MMP)

Mitochondrial membrane potential was assessed using TMRE (tetramethylrhodamine ethyl ester; 200 nM, Sigma-Aldrich) as a fluorescent probe. HCMs were incubated with the probe at 37 °C for 30 min, followed by fluorescence imaging using a KEYENCE All-in-One microscope (KEYENCE, Osaka, Jaapn). Fluorescence intensity was quantified using NIH ImageJ software (v1.52).

### 4.9. Mitochondrial Stress Analysis (Seahorse Assay)

Mitochondrial function was evaluated using the Seahorse XFe24 Extracellular Flux Analyzer (Agilent Technologies), as previously described [83,84]. HCMs were seeded in 6-well plates and treated with either 20% *v*/*v* ascitic fluid or conditioned medium prior to assay. Oxygen consumption rate (OCR) was monitored in real time following the sequential injection of 1 μM oligomycin, 2 μM FCCP, 1 μM rotenone, and 5 μM antimycin A. Data were used to calculate ATP-linked respiration, maximal respiration, proton leak, and spare respiratory capacity.

### 4.10. Glycolytic Stress Test

Glycolytic flux was measured by determining the extracellular acidification rate (ECAR) using the Seahorse XFe24 system with XF24 FluxPaks (Agilent Technologies, Santa Clara, CA, USA). HCMs were pre-cultured in 6-well plates and then transferred to XF base medium containing 200 mM L-glutamine and 5 mM HEPES. The sensor cartridge was hydrated with XF calibrant overnight. Sequential injections included glucose (10 mM), oligomycin (1 μM), a combination of rotenone (1 μM) and antimycin A (5 μM), and finally 2-deoxyglucose (50 mM). ECAR changes reflected glycolytic capacity and compensation mechanisms in response to mitochondrial dysfunction.

### 4.11. RNA Immunoprecipitation (RIP)

To assess AGO2-RNA interactions, HCMs were incubated with 8 μM TAMRA-labeled ACHF antisense RNA (synthesized by Tsukuba Oligo Service, Ushiku, Japan) for 48 h. Cells were lysed in RIPA buffer supplemented with RiboLock RNase Inhibitor. Lysates (40 μg) were pre-cleared with Protein A/G agarose (Santa Cruz Biotechnology), then incubated with anti-AGO2 antibody (clone 4G8, Wako, Osaka, Japan) at 4 °C for 90 min. Immunoprecipitates were collected, washed, and resuspended in DEPC-treated water. Fluorescence intensity was measured using the Qubit 4 Fluorometer (Thermo Fisher Scientific, Waltham, MA, USA).

### 4.12. Sequence Homology and siRNA Prediction

Homology between ACHF and human genomic sequences was evaluated using the BLAST algorithm (https://blast.ncbi.nlm.nih.gov, accessed on 24 September 2020). For siRNA target prediction, the siDirect v2.1 tool (https://sidirect2.rnai.jp/, accessed on 25 February 2025) was employed to assess potential binding sites within the ATP5A transcript.

### 4.13. Statistical Analysis

Statistical evaluations were performed using GraphPad InStat software (v3.1; GraphPad Software, La Jolla, CA, USA). One-way analysis of variance (ANOVA) was used for group comparisons. All values are presented as mean ± standard deviation (SD) from at least three independent experiments. Statistical significance was set at *p* < 0.05 (two-tailed).

## 5. Conclusions

This study demonstrated that a short sequence that is homologous between the SARS-CoV-2 genome and the human ATP5A gene can act in an siRNA-like manner to suppress ATP5A expression. This suppression resulted in impaired mitochondrial energy metabolism, disrupted cardiomyocyte maturation, and increased apoptosis—factors that may collectively contribute to the development of heart failure. To establish the physiological relevance of these findings, further validation using in vivo models and clinical samples from SARS-CoV-2-infected patients is required. Such investigations will be critical in determining whether viral RNA fragments play a causative role in COVID-19-associated cardiac injury.

## Figures and Tables

**Table 1:** Homology between the *ATP5A* gene and SARS-CoV-2 genome.

Gene Symbol	Accession Number	1st Base	Sequence
*Human ATP5A*	M37104.1	400	AAT AAA CAT GCA TTT CAT A
*Mouse ATP5j*	NM_001358500.1	626	AAT AAA CAT TCA TTT CAC A
*Rat ATP5PF*	NM_053602.2	552	AAT AAA CAT TCA CTT CAC A
*HKU1B.9 strain SC2521*	ON461763.1	19314	AAT AAA CAT GCA TTT CAT A
*SARS-CoV-2/Wuhan*	NC_045512.2	19303	AAT AAA CAT GCA TTC CAC A
*SARS-CoV-2/USA/WA* *-PHL-001844*	OV278871.2	19303	AAT AAA CAT GCA TTC CAA C
*SARS-CoV-2/Japan/sb_ncgms* *_cov_2_02761/2022*	BS012486.1	19273	AAT AAA CAT GCA TTT CAT A
*SARS-CoV-2/USA/LA-CDC* *-STM-94SR8FMQV/2022*	OP399780.1	19283	AAT AAA CAT GCA TTT CAT A

Red letters indicate a different base from those in human *ATP5A.*

**Table 2:** PCR primers and antibodies.

RT-PCR Primers			
Gene	ID	Upper	Lower
*ATP5A*	NM_001101.3	GGACTTCGAGCAAGAGATGG	AGCACTGTGTTGGCGTACAG
*Myomesin*	AJ621424.1	GATACAGCTCAGTACCGGGC	CTTTGTTGGCCTCCAAGCAC
*Myogenin*	NM_002479.6	TACCAGGAACCCCGCTTCTA	GTGATGCTGTCCACGATGGA
*Troponin T*	S69208.1	TTCGATGACATCCACCGCAA	TTTCAGCTTCGCCATCAGGT
**Antibodies**			
Target	Cat No.	Company	
IRF3	ab68481	Abcam, Waltham, MA, USA	
IRF7	22392-1-AP	Proteintech, Rosemont, IL, USA	
ATP5A	ab110273	Abcam, Waltham, MA, USA	
β-actin	sc-47778	Santa Cruz Biotechnologies, Santa Cruz, CA, USA	

RT-PCR, reverse transcription-polymerase chain reaction.

## Data Availability

Data is contained within the article.

## Abbreviations

SARS-CoV-2: severe acute respiratory syndrome coronavirus 2; AGO2, argonaute 2; siRNA, short interfering RNA; COVID-19, coronavirus disease 2019; ACE2, angiotensin-converting enzyme 2; ssRNA, single-stranded RNA; ETC, electron transport chain; ORF, open reading frame; ACHF, ATP5A-CoV Homologous Fragment; S, sense; AS, antisense; HCM, human cardiomyocyte; DAMP, damage associated molecular patterns; IFN, interferon; TLR, toll-like receptor; IRF, interferon regulatory factors; ECAR, extracellular acidification rate; 4HNE, 4-hydroxynonenal; MMP, mitochondrial membrane potential; SDS-MYL1, sulfate-soluble myosin light chain 1; MYH, myosin heavy chain; ANP, atrial natriuretic peptide; RISC, RNA-induced silencing complex; EIPA, 5-[N-ethyl-N-isopropyl] amiloride; ncRNA, non-coding RNA; lncRNA, long non-coding RNA; OXPHOS, oxidative phosphorylation; miRNA, microRNA; ROS, reactive oxygen species.
